# Implementation of new Westgard rules suggested by the Westgard Advisor software for five immunological parameters

**DOI:** 10.11613/BM.2025.010701

**Published:** 2024-12-15

**Authors:** Lisa Cristelli, Francesca Occhipinti, Daniel Tumiatti, De Luisi Antonia, Erika Jani, Massimo Daves

**Affiliations:** 1 Clinical Pathology Laboratory, Hospital of Silandro, Silandro, Italy; 2 Clinical Biochemistry Laboratory, Provincial Hospital of Bolzano (SABES-ASDAA), Bolzano, Italy

**Keywords:** laboratory organization and management, quality control, validation, evaluation, Westgard rules

## Abstract

**Introduction:**

Knowledge and systematic evaluation of analytical errors is the task of internal analytical quality control management. The aim of this study was to assess whether the Westgard rules proposed by Bio-Rad’s Westgard Advisor software are more efficient in the monitoring of analytical performance than those previously in use.

**Materials and methods:**

The study was carried out on the nephelometer Atellica NEPH630 (Siemens Healthineers, Erlangen, Germany). Five parameters were chosen: serum immunoglobulin A (IgA), alpha 1 - antitrypsin (AAT), prealbumin, lipoprotein (a) (Lp(a)) and ceruloplasmin. The study was divided into 4 phases (A, B, C, D): phase A - old rules used (1_3s_, R_4s_ and 2_2s_); phase B - first introduction of new rules (30 days), (1_3s_/2_2s_ for IgA; 1_3s_/2_2s_/R_4s_/4_1s_/10_x_ for the remaining parameters); Phase C - second intervention (after 60 days) 1_3s_/2_2s_/R_4s_/4_1s_ for IgA and Lp(a), 1_3s_/2_2s_/R_4s_/4_1s_/8_x_ for prealbumin and ceruloplasmin and 1_3s_/2_2s_/R_4s_/4_1s_/10_x_ for AAT; and Phase D - values at the end of the study (1_3s_ for IgA, 1_3s_/2_2s_/3_2s_/R_4s_/3_1s_/12_x_ for AAT and ceruloplasmin, 1_3s_/2_2s_/R_4s_/4_1s_/8_x_ for prealbumin and 1_3s_/2_2s_/R_4s_/4_1s_/10_x_ for Lp(a).

**Results:**

At the end of the study the coefficient of variation (CV%), bias (%) and sigma for IgA were 2.55%, - 1.09% and 5.33, respectively; for AAT 3.88, - 2.21 and 3.25; for prealbumin 3.99, - 0.14 and 2.95; for Lp(a) 8.02, - 0.34 and 3.81; for ceruloplasmin 2.48, - 3.65 and 3.49.

**Conclusions:**

By using newly suggested rejection rules, we did not observe an improvement in monitoring of analytical performance.

## Graphical abstract

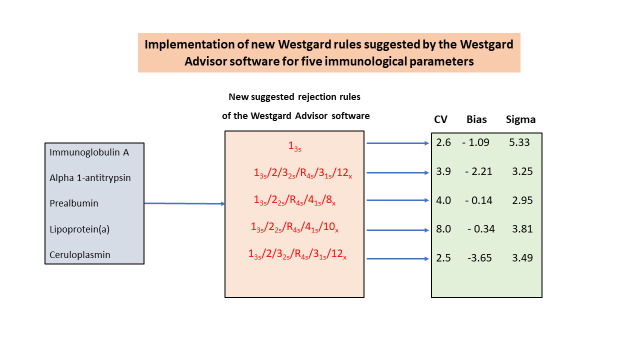

## Highlights

• Each clinical laboratory must develop its own internal quality control strategy

• Specific rules are used to understand and categorize the results of internal quality control measurements

• We assessed new Westgard rules proposed directly by Bio-Rad’s Westgard Advisor software against those previously in use

• By using newly suggested rejection rules for the studied parameters, the analytical quality was not significantly improved

## Introduction

Laboratory medicine plays an increasingly important role in today’s healthcare system. Laboratory analyses are an integral part of clinical diagnosis. The analytical process must focus on patient care and safety, so laboratory reports must be analytically correct and reliable (*1*). Unfortunately, analytical errors can lurk anywhere in the complex process of analysis and the consequences can represent potential harm to the patient. For this reason, the laboratory must ensure that the results are reliable and accurate (*2*). Comprehensive knowledge and systematic evaluation of analytical laboratory errors are therefore essential. This is precisely the task of internal analytical quality control (IQC) management. It helps to recognize potential errors in the analytical process at an early stage (*3*). Each clinical laboratory must develop its own IQC strategy to minimize the likelihood of patient harm due to an out-of-control system (*4*). To evaluate the success of an IQC strategy for individual measurement parameters, the following key figures are used in relation to the analytical concentration: coefficient of variation (CV), bias and sigma (*5*). At least two different concentration levels of the IQC should be measured. One level is usually within the reference values, while the other level is in the pathological range.

In clinical routine, every laboratory test is subjected to a daily test program and the analytical quality is checked with an appropriate statistical analysis. The accuracy and precision of the results are thus monitored (*6*). Recently Sonntag *et al.* reported some interesting considerations on the possibility of using two or three levels of controls, concluding that the laboratory must evaluate which strategy is best for its needs (*7*). Specific rules are used to understand and categorize the results of IQCs measurements and to assess the possibility of accepting the validity of the analytical session. These rules are the “Westgard rules” that can be used to interpret the results on the Levey-Jennings control chart and these rules as predefined control limits are available in commercially specific software (*5*, *8*). Our laboratory uses the Unity real time (URT) software (Bio-Rad Laboratories Inc., Hercules, USA) for the management and evaluation of IQCs. Internal quality control values are automatically entered into the software, which draws up ‘Levey-Jennings’ control charts for each individual analyte and control level. The aim of this retrospective study was to assess whether the Westgard rules proposed by Bio-Rad’s Westgard Advisor software are more efficient than those previously in use. Westgard Advisor is a function in the URT that generates optimal Westgard rules for selected parameters and recommends their application. The software is useful for recognizing tests that need to be improved and those that regularly achieve the required quality. The objective was to observe whether these newly proposed rules contribute to improved analytical quality and whether they promote IQC management. Specifically, we hypothesized that the Westgard Advisor software creates new specific Westgard rules for five selected immunological parameters that guarantee the highest possible quality of the tests.

## Material and methods

The study was carried out on the nephelometer Atellica NEPH630 (Siemens Healthineers, Marburg, Germany). The analytical performance was evaluated over a period of 2 months (from 8 June to 8 August 2022). Five immunological parameters measured on the nephelometer were chosen for the study: serum immunoglobulin A (IgA), alpha 1-antitrypsin (AAT), prealbumin, lipoprotein (a) (Lp(a)) and ceruloplasmin. These parameters were chosen because they were the ones with a lower analytical performance than the other parameters measured with the nephelometer. All tests were performed following the manufacturer’s instructions. The IQC from the manufacturer Bio-Rad is immediately loaded onto the nephelometer once a day, usually at 8:00 a.m., and measured. Two different control levels (Level 1 and Level 3) are used for the IQC. The results of the IQC are automatically transferred to the URT Levey-Jennings chart and can be viewed and validated. On 8 June 2022, the old rejection rules (1_3s_, R_4_s and 2_2_s rule) for the study parameters, were replaced by the suggested new rejection rules of the Westgard Advisor software (Table 1). The purpose of the 1_3s_ rule is to indicate a random error or the start of a systematic error. In contrast to the 1_2s_ rule, the 1_3s_ rule is not a warning rule, but a rejection rule. The R_4s_ rule recognizes random errors. If there is a difference of at least ± 4 standard deviations (SD) between the control limits, the rule is considered to have been violated. The run must be rejected. This is also referred to as an “out-of-control condition.” The 2_2s_ rule is designed to detect only systematic errors. The 2_2s_ rule is violated if two consecutive IQC results lie outside the ± 2SD, *i.e.*, on the same side of the mean. A violation of this rule indicates an out-of-control condition. The new rejection rules proposed by the Westgard Advisor software during the study were: 2/3_2s_, 4_1s_, 3_1s_, 10_x_, 8_x_ and 12_x_.

**Table 1:** New suggested rejection rules of the Westgard Advisor software during the study

**Test**	**Control level that triggered the new rule**	**Rejection rules before the start of the study**	**New rejection rules from 08 June 2022**	**New rejection rules from 8 July 2022**	**New rejection rules from 8 August 2022**
IgA (g/L)	Level 3	1_3s_ /R_4s_/2_2s_	1_3s_/2_2s_	1_3s_/2_2s_/R_4s_/4_1s_	1_3s_
AAT (g/L)	Level 3	1_3s_ /R_4s_/2_2s_	1_3s_/2_2s_/R_4s_/4_1s_/10_x_	1_3s_/2_2s_/R_4s_/4_1s_/10_x_	1_3s_/2/3_2s_/R_4s_/3_1s_/12_x_
Prealbumin (g/L)	Level 1	1_3s_ /R_4s_/2_2s_	1_3s_/2_2s_/R_4s_/4_1s_/10_x_	1_3s_/2_2s_/R_4s_/4_1s_/8_x_	1_3s_/2_2s_/R_4s_/4_1s_/8_x_
Lp(a) (g/L)	Level 1	1_3s_ /R_4s_/2_2s_	1_3s_/2_2s_/R_4s_/4_1s_/10_x_	1_3s_/2_2s_/R_4s_/4_1s_	1_3s_/2_2s_/R_4s_/4_1s_/10_x_
Cp (g/L)	Level 3	1_3s_ /R_4s_/2_2s_	1_3s_/2_2s_/R_4s_/4_1s_/10_x_	1_3s_/2_2s_/R_4s_/4_1s_/8_x_	1_3s_/2/3_2s_/R_4s_/3_1s_/12_x_
IgA - immunoglobulin A. AAT - alfa 1-antitrypsin. Lp(a) - lipoprotein (a). Cp - ceruloplasmin.					

The 2/3_2s_ rule is a variant of the 2_2s_ rule and indicates the presence of a systematic error. The 4_1s_ rule is considered violated if four consecutive control results are greater than the ± 1SD and are on the same side of the mean. A violation of these rules usually indicates a tendency towards systematic errors; the analyzer is in an out-of-control condition. The 3_1s_ rule is considered violated if three consecutive control results are greater than the ± 1SD and are on the same side of the mean. A violation of this rule usually indicates a tendency towards a systematic error. The analysis system is in an out-of-control condition. The 10_x_ rule is sensitive to systematic errors and is applied if 10 consecutive control results are on one side of the mean value (out of control condition). The 8_x_ rule is similar to the 10_x_ rule. This rule is sensitive to systematic errors. In contrast to the 10_x_ rule, this rule only applies if there are eight consecutive control results on one side of the mean value (out of control condition). The 12_x_ rule is similar to the 10_x_ and 8_x_ rule. This rule is sensitive to systematic errors. In contrast to the 10_x_ rule, this rule only applies if twelve consecutive control results are on the same side of the mean value (out of control condition) (*9*). At the beginning of the study, CV, bias and sigma were recorded by the URT software for the five parameters. This provided an overview of how precisely the device worked with the rejection rules (1_3s_, 2_2s_ and R_4s_ rule) that have been used for years in our laboratory. The study was then divided into 4 phases (phase A: old rules used; phase B: first introduction of new proposed rules (30 days); phase C: second intervention (after 60 days); phase D: shows the values at the end of the study), including the update of the Westgard rules suggested by the Westgard Advisor software of the Bio-Rad company. The Westgard Advisor software only creates certain rules for a test if the minimum data requirements are met. The rules for a test are created only when the minimum number of laboratory data points (at least 20), the minimum number of individual values of the comparison group (at least 100) and the minimum number of comparison group laboratories (at least 5) are available. The minimum numbers of all three data requirements were used for the study. This means that the software generated the new rejection rules for the five laboratory parameters based on 20 laboratory data points, 100 values of the comparison group and 5 comparison laboratories.

These new rules were automatically formulated by the program in relation to the control level showing the worst performance in accordance with an analysis provided by the software itself. The new rules proposed by the software were applied to both control levels (Table 1).

### Statistical analysis

The re-elaborations were carried out twice during the entire study period. The following statistical coefficients were monitored at the different reworking stages to assess any improvement in the analytical quality of the five parameters evaluated: CV%, bias% and sigma. In URT, the CV% for all five parameters at two concentration levels in the four different phases of the study was calculated with the following formula: CV% = (SD/mean) x 100%, where mean represents the mean of our own IQC controls and SD is the corresponding standard deviation of our own IQC controls. The bias was calculated with the following formula: bias = [(mean-group mean)/group mean] x 100, where group mean is the mean of the homogeneous group (other laboratories that use the same method). The sigma was calculated as: Sigma = (TEa – bias)/SD, where TEa is the allowable total error. The allowable total error of the five parameters was automatically recorded and calculated by the software URT: TEa = z score x (imprecision goal,%) + [bias goal,%].

## Results

The different phases represent the statistical development and progression of the CV over the entire duration of the study (Table 2). At first glance, it is easy to see that the CV has improved noticeably in 4 out of 5 parameters (excluded for Lp(a)) at the end of the study). In phase B, a minimal deterioration of the mean CV can be recognized. The minimal deterioration in phase B can be explained by the fact that the activation of the new rejection rules of Westgard Advisor meant that more rejection rules had to be observed. However, in phase C, after the second month of activating these rules, it became apparent that the CV of the parameters was slowly but steadily improving again. In phase D at the end of the study, the mean CV was 4.2%. This is the same value as in phase A, *i.e.*, before the start of the study. The mean CV value therefore showed no change or improvement at the end of the study. A “worsening” of the bias is already recognizable in phase B. This deterioration can be seen throughout the entire study. This can be explained by the fact that the values first had to stabilize due to the many new rejection rules that had to be observed. Only two parameters were no longer as close to the mean value at the end of the study as before the start of the study. While the mean bias in phase A, *i.e.,* before the start of the study, was 1.28%, it was - 1.49% by the end of phase D. It can also be seen that the values of the parameters before the start of the study were still above the mean value, while after the study the values were below the mean value. At first glance, it is easy to see that sigma improved slightly in 3 out of 5 parameters (IgA, Ceruloplasmin, AAT). In phase B, a minimal deterioration of sigma is recognizable. While the mean sigma in phase A, *i.e.,* before the start of the study, was 3.75, it was 3.77 in phase D (Table 2).

**Table 2:** Bias, coefficient of variation and sigma in the different observation phases of the study

	**Phase A**			**Phase B**			**Phase C**			**Phase D**		
**Test**	**CV**	**Bias**	**Sigma**	**CV**	**Bias**	**Sigma**	**CV**	**Bias**	**Sigma**	**CV**	**Bias**	**Sigma**
IgA	2.9	0.07	5.03	3.1	- 0.13	4.75	2.6	- 0.96	5.20	2.6	-1.09	5.33
AAT	5.1	0.63	3.09	3.5	- 3.25	4.20	3.9	- 2.30	3.21	3.9	- 2.21	3.25
Prealbumin	4.3	1.71	3.62	5.3	0.94	2.88	4.0	0.26	2.90	4.0	- 0.14	2.95
Lp(a)	5.0	2.29	3.86	7.2	0.20	4.71	8.1	- 0.56	3.76	8.1	- 0.34	3.81
Cp	3.6	1.72	3.15	2.6	- 2.19	1.96	2.6	- 3.64	3.38	2.5	- 3.65	3.49
IgA - immunoglobulin A. AAT - alfa 1-antitrypsin. Lp(a) - lipoprotein (a). Cp – ceruloplasmin. CV - coefficient of variation. Phase A - old rules used. Phase B - first introduction of new rules. Phase C - second intervention. Phase D - end of the study.												

## Discussion

The study objective was to assess whether the use of the new Westgard rules suggested by Westgard Advisor software could lead to a better analytical performance monitoring of the five selected parameters, detecting shifts more effectively. This would have been visible in the fact that the values of sigma would have increased, while the values of CV and Bias would have decreased. These desirable results could not be achieved for all five parameters. It can therefore be said that the suggested rejection rules of Westgard Advisor and the associated interventions carried out did not lead to significant improvement in the analytical performance monitoring of the five study parameters. However, inhomogeneous improvements in the three statistical values were observed over the entire duration of the study. In the first observation after 30 days (phase B), CV improved in only 2 of 5 parameters (AAT and ceruloplasmin) bias and sigma. In the second observation phase, after 60 days (phase C), the values of the five study-relevant parameters then showed an initial inhomogeneous improvement. Sigma improved in 3 out of 5 parameters. The CV improved in 4 of 5 parameters and the bias showed an improvement in 2 of the 5 parameters. In the third observation phase (phase D), sigma improved again in 3 of the 5 parameters. The CV and bias also improved again slightly. The CV showed an improvement in 4 of 5 parameters and bias in 2 of 5 parameters. Westgard Advisor suggested a large number of rejection rules for 4 of the 5 study parameters. It was difficult to always adhere to these. This was the 10_x_, 8_x_ and 12_x_ rule. Adhering to these rules proved to be particularly difficult, as it happened relatively frequently that some values were 8, 10 or 12 times above or below one side of the mean value. The duration of the study was 2 months. This is a short period of time and longer study duration would certainly be recommended to confirm our results. However, the study was a complete success in other aspects. The activation of the many rejection rules and the posting of at least one of these rejection rules *per* day led to a sensitization of the laboratory technicians regarding the IQC. The laboratory technicians developed a better approach to IQC, and it is important because IQC can often fail mainly because they are not handled correctly, and the right approach is lacking. Because of the study, the working behavior of the laboratory technicians changed noticeably for the better. Much more attention was paid to the correct handling of the IQC. The study hypothesis could not be successfully achieved. This means that the study was unfortunately unable to produce the desired results.

A limitation of the study to be taken into account is that different lot of reagents were used during the evaluation period, which could partly be responsible for the observed results.

In conclusion, by using the Westgard Advisor software and the associated newly suggested rejection rules for the five study parameters, we could not observe an improvement in monitoring analytical performance; nevertheless, there were inhomogeneous improvements in the three statistical values. Our results can be compared to those obtained by Karnutsch *et al*. in a study that assessed whether the use of different Westgard rules could influence the IQC strategy with a reduction in the risk management index, which represents an estimate of the probability of patient harm divided by the acceptable probability of harm. Also in this study, the authors could not demonstrate that use of different and stricter rejection rules would lead to an improved in the analytical performance (*10*). Very recently, Åsberg and Bolan reported on the use of receiver operating characteristic curve analysis to analyze the diagnostic accuracy of different quality control rule sets in the ability to discriminate the presence or absence of systematic error (*11*). This innovative approach emphasizes the importance of the search for more efficient control strategies to ensure greater safety in clinical laboratory test results.

## Data Availability

The data generated and analyzed in the presented study are available from the corresponding author on request.
